# Social Epidemiology of Early Initiation of Electronic and Conventional Cigarette Use in Early to Middle Adolescents

**DOI:** 10.31586/jbls.2024.1038

**Published:** 2024-09-04

**Authors:** Shervin Assari, Hossein Zare, Payam Sheikhattari

**Affiliations:** 1 Charles R. Drew University of Medicine and Science, Los Angeles, CA, United States; 2 Johns Hopkins Bloomberg School of Public Health, Baltimore, MD, United States; 3 School of Business, University of Maryland Global Campus (UMGC), Adelphi, MD, United States; 4 Morgan State University, Baltimore, MD, United States

**Keywords:** Tobacco, Electronic Cigarette, Combustible Tobacco, Adolescents, Youth, Social Determinants

## Abstract

**Background:**

Early initiation of tobacco use among adolescents is a significant public health concern. While there is extensive research on overall tobacco use, much of it focuses on initiation in late adolescence, uses cross-sectional designs, and lacks specific exploration of electronic versus conventional cigarette use. This study aims to investigate social determinants influencing the early initiation of electronic and conventional cigarette use among U.S. adolescents.

**Methods:**

We utilized data from the Adolescent Brain Cognitive Development (ABCD) study, which follows a cohort of tobacco-naïve children from age nine through age 16. The social determinants examined included household income, parental education, financial difficulties, racial/ethnic minority status, family structure, neighborhood income, and gender minority status. Structural equation models were employed to assess associations between these determinants and early initiation of electronic and conventional cigarette use.

**Results:**

Male gender was associated with a higher likelihood of conventional cigarette use, while the risk of early initiation of electronic cigarette use was similar across genders. White adolescents were at a higher risk of conventional cigarette use; however, the risk for electronic cigarette use was comparable across White and non-White groups. Financial difficulties were linked to an increased likelihood of early initiation of conventional cigarette use but not electronic cigarette use. Higher household income was associated with a reduced risk of initiating conventional cigarettes but did not significantly impact electronic cigarette use. Adolescents from married families were less likely to initiate electronic cigarette use. No significant effects were found for parental education or neighborhood income on the initiation of either type of cigarette use. Age did not significantly affect the initiation of either cigarette type, and gender minority status was marginally associated with early initiation of conventional cigarette use.

**Conclusions:**

The social patterning of electronic cigarette use differs from that of conventional cigarette use, suggesting that distinct tobacco products do not pose a uniform risk across all adolescents. This study underscores the importance of tailored prevention efforts that address the unique challenges associated with early initiation of electronic and conventional cigarette use among adolescents. The differential risk factors identified suggest targeted prevention strategies for conventional cigarette use, focusing on financial difficulties, household income, and gender-specific interventions. In contrast, prevention efforts for electronic cigarette use may require broader, more inclusive approaches that address all adolescents, regardless of their background. Comprehensive universal screening for electronic cigarette use and targeted screening for conventional cigarette use among adolescents are recommended.

## 1. Introduction

Early initiation of electronic and conventional cigarette use among adolescents is a growing public health concern [1]. However, the social determinants influencing the use of newer tobacco products remain inadequately understood. Existing research predominantly focuses on adults or older adolescents, traditional products, and employs cross-sectional designs, leaving a gap in our understanding of the factors that contribute to the early initiation of various tobacco products during early to middle adolescence [2]. This gap is particularly pronounced in studies examining the predictive utility of baseline social determinants for future conventional and electronic cigarette use initiation.

Limited studies on early to middle adolescents present inconsistent findings regarding the impact of various social determinants, such as household income, parental education, financial difficulties, racial and ethnic minority status, family structure, gender minority status, and immigration status [3]. These inconsistencies suggest that the influence of these factors on tobacco use initiation may be complex and multifaceted, potentially differing between electronic and conventional cigarette use.

Given these complexities, it is crucial to investigate whether the social determinants of electronic cigarette use initiation differ from those of conventional cigarette use initiation. This differentiation has policy implications, as screening and diagnosis of electronic and conventional cigarette use may target different populations. Given their risk profiles, different subgroups of adolescents should be prioritized. Understanding these differences can inform targeted or even universal prevention and intervention strategies [4].

The Adolescent Brain Cognitive Development (ABCD) Study [5–10] provides a unique opportunity to address these gaps in existing knowledge. By following a large cohort of tobacco-naïve children from the age of nine through early and middle adolescence, the ABCD study allows for a comprehensive examination of the prospective social determinants of tobacco use initiation across various products. This longitudinal design is particularly advantageous for capturing the dynamic nature of adolescents who were tobacco-free and could show the evolving influences of social determinants over time as they uptake tobacco.

## 2. Aims

The aim of this study, primarily exploratory in nature, is to elucidate the differential impacts of household income, parental education, financial difficulties, racial and ethnic minority status, family structure, gender minority status, and immigration status on the early initiation of electronic and conventional cigarette use during early to middle adolescence. By leveraging the robust dataset provided by the ABCD study, the results may contribute to a deeper understanding of the variation in social determinants of adolescent tobacco use initiation, based on tobacco products.

## 3. Methods

### 3.1. Study Design and Sample

We performed a secondary analysis of data from the Adolescent Brain Cognitive Development (ABCD) study, a national longitudinal investigation that includes a diverse sample of pre-adolescent children transitioning to adolescence. The ABCD study is an ongoing cohort that began between 2016 and 2018 (baseline). The currently available data include assessments of youth who are 16 years old, collected in 2022. The ABCD study is characterized by its longitudinal, national, large, and diverse samples in terms of race, socioeconomic position (SEP), and geography. Participants were primarily recruited through schools. Our analytical sample consisted of pre-adolescents who reported no tobacco use at baseline. Participants included both native and U.S.-born adolescents from various racial and ethnic backgrounds.

### 3.2. Ethics

The ethical aspects of the ABCD study were approved by the Institutional Review Board (IRB) of the University of California, San Diego (UCSD). All adolescent participants provided assent, and their parents signed informed consent forms.

### 3.3. Predictors

#### Family Income:

Family income was measured on a scale of 1 to 10, with higher scores indicating higher income. The total combined family income for the past 12 months was reported using the following categories: 1 = less than $5000; 2 = $5000+; 3 = $12,000+; 4 = $16,000+; 5 = $25,000+; 6 = $35,000+; 7 = $50,000+; 8 = $75,000+; 9 = $100,000+; and 10 = $200,000+. This variable was treated as a continuous measure.

#### Parental Educational Attainment:

Participants reported the highest grade or level of school completed or the highest degree received, using a scale ranging from 0 (never attended/Kindergarten only) to 21 (doctoral degree). This variable was treated as an interval measure, with higher scores indicating higher parental educational attainment.

#### Family Structure:

Parents reported the number of parents in the household and their marital status. This variable was categorized as 0 for unmarried and 1 for married.

#### Neighborhood Income:

Using zip code data, median family income in the zip code was recorded and divided by 5000 for easier interpretation of beta coefficients.

#### Race/Ethnicity:

Parents reported the race/ethnicity of the participants, categorized as racial/ethnic minority groups (reference category) and non-Latino Whites.

#### Financial Difficulties:

Financial difficulties were measured using seven items assessing whether the family experienced issues such as needing food but being unable to afford it, lack of telephone service, inability to pay rent or mortgage, eviction, utility shut-offs, and inability to afford medical or dental care. Responses were binary (0 or 1), and a total score was calculated with a range of 0 to 7, with higher scores indicating greater financial difficulties.

### 3.4. Outcome

#### Conventional and Electronic Cigarette Use:

Tobacco use was assessed every six months using the iSay Sipping Inventory for recent or first experimentation with nicotine products. At baseline, adolescents reported lifetime use of substances through a web-based Timeline Follow-Back (TLFB) interview. Follow-up assessments were conducted at six-month intervals. For this analysis, conventional and electronic cigarette use initiation were the primary outcomes, defined as new onset of use captured six months or later after baseline.

### 3.5. Data Analysis

We conducted the analysis using Stata 18.0. Structural equation modeling (SEM) was used with two outcomes in the same model: conventional and electronic cigarette use initiation. Covariates included sex and age. Predictors were household income, parental education, financial difficulties, race/ethnicity, family structure, gender minority status, and neighborhood income.

## 4. Results

As shown by Figure, male gender was associated with higher conventional cigarette use only; both genders were equally at risk of early initiation of electronic cigarette use. White adolescents were at higher risk of conventional cigarette use, while the risk for electronic cigarette use was similar across White and non-White groups. Financial difficulties were associated with a higher likelihood of early initiation of conventional cigarette but not electronic cigarette. Household income was a significant factor for reduced initiation of conventional but not electronic cigarette use. Adolescents from married families were less likely to initiate electronic cigarette use. There was no effect of immigration status, parental education, or neighborhood income on the initiation of either type of cigarette use. Age did not show a significant effect on the initiation of either type of cigarette use. Gender minority status was marginally associated with early initiation of conventional cigarette.

Table 1 shows the results of the structural equation model (SEM) for conventional and electronic cigarette use. For conventional cigarette use, neighborhood income per $50,000 was not significantly associated (B = −0.006, SE = 0.011, 95% CI: −0.027 to 0.015, p = 0.548). However, higher household income was associated with lower conventional cigarette use (B = −0.050, SE = 0.015, 95% CI: −0.080 to −0.020, p = 0.001). Being Non-Latino White was positively associated with conventional cigarette use (B = 0.031, SE = 0.011, 95% CI: 0.010 to 0.051, p = 0.004). Financial difficulty was also positively associated with higher conventional cigarette use (B = 0.032, SE = 0.010, 95% CI: 0.012 to 0.052, p = 0.002). Age per 10 years was not significantly associated with conventional cigarette use (B = 0.007, SE = 0.009, 95% CI: −0.011 to 0.025, p = 0.466), but males were more likely to use conventional cigarettes (B = 0.025, SE = 0.009, 95% CI: 0.007 to 0.043, p = 0.006). Living in a married household was not significantly associated with conventional cigarette use (B = −0.015, SE = 0.011, 95% CI: −0.037 to 0.007, p = 0.170). The association between gender minority status and conventional cigarette use was marginally non-significant (B = 0.017, SE = 0.009, 95% CI: −0.001 to 0.035, p = 0.062). Parental education was not significantly associated with conventional cigarette use (B = 0.008, SE = 0.012, 95% CI: −0.016 to 0.032, p = 0.517). The intercept for conventional cigarette use was significant (B = 0.131, SE = 0.066, 95% CI: 0.001 to 0.261, p = 0.048).

For electronic cigarette use, neighborhood income per $50,000 was not significantly associated (B = −0.005, SE = 0.011, 95% CI: −0.026 to 0.016, p = 0.669), and household income showed no significant association with electronic cigarette use (B = 0.000, SE = 0.016, 95% CI: −0.031 to 0.032, p = 0.978). Non-Latino White status was not significantly associated with electronic cigarette use (B = 0.007, SE = 0.011, 95% CI: −0.013 to 0.028, p = 0.488). Financial difficulty was also not significantly associated with electronic cigarette use (B = 0.011, SE = 0.010, 95% CI: −0.009 to 0.031, p = 0.268). Age per 10 years did not show a significant association (B = −0.012, SE = 0.009, 95% CI: −0.030 to 0.006, p = 0.176), and being male was not significantly associated with electronic cigarette use (B = 0.013, SE = 0.009, 95% CI: −0.005 to 0.031, p = 0.159). However, living in a married household was associated with lower electronic cigarette use (B = −0.034, SE = 0.011, 95% CI: −0.056 to −0.012, p = 0.003). Gender minority status was not significantly associated with electronic cigarette use (B = −0.004, SE = 0.009, 95% CI: −0.022 to 0.014, p = 0.654), and parental education also showed no significant association (B = 0.010, SE = 0.012, 95% CI: −0.014 to 0.035, p = 0.399). The intercept for electronic cigarette use was not significant (B = 0.034, SE = 0.066, 95% CI: −0.096 to 0.163, p = 0.609).

## 5. Discussion

Our longitudinal study investigated the social determinants of early initiation of electronic and conventional cigarette use among U.S. adolescents using data from the ABCD study. The findings reveal distinct patterns of influence that differ between conventional and electronic cigarettes and vary based on various social determinants, underscoring the complexity of the social patterning of early tobacco use behaviors in the U.S. Overall, our findings highlight that electronic cigarette use does not follow the same social patterning as conventional cigarette use. Electronic cigarettes may pose a universal risk, affecting adolescents across all races, socioeconomic statuses, and genders. This emerging trend calls for a reevaluation of prevention and intervention strategies to address the unique challenges posed by electronic cigarettes.

Firstly, financial difficulty was associated with a higher likelihood of initiating conventional cigarette use, but it did not appear to influence electronic cigarette use. This suggests that financial stressors may drive adolescents toward traditional tobacco products, possibly due to their perceived affordability or accessibility. Financial difficulty reflects poverty, a known risk factor for conventional cigarette use. Poverty is not necessarily a risk factor for electronic cigarette use, which is less affordable for low-income populations. In addition, conventional cigarettes might be more readily available or perceived as cheaper than electronic alternatives in economically strained environments, leading adolescents under financial stress to opt for these products. Furthermore, conventional cigarettes might be more accepted by the poor as an effective strategy for stress relief, aligning with the coping mechanisms of financially distressed populations.

Household income emerged as a significant factor, whereas parental education did not substantially impact the initiation of either electronic or conventional cigarette use. Despite research on SES suggesting that education may have a larger effect on behaviors than income, we did not find such results. This indicates that financial resources, rather than educational background, play a crucial role in adolescent tobacco use behaviors. Higher household income may provide adolescents with greater access to various tobacco products, while the lack of significant influence from parental education suggests that educational attainment alone is insufficient to deter adolescent tobacco use. These findings highlight the importance of economic conditions in shaping adolescent health behaviors.

Family structure also showed a differential impact observable for one product type but not the other. Adolescents from married families were less likely to initiate electronic cigarette use, highlighting the protective effect of a stable family environment against newer forms of tobacco products. A stable family structure may provide better supervision, emotional support, and communication about the risks of tobacco use, thereby reducing the likelihood of adolescents experimenting with electronic cigarettes. This protective effect underscores the critical role of family dynamics in preventing tobacco use initiation.

Regarding racial and ethnic differences, White adolescents were at higher risk of initiating conventional cigarette use compared to their peers, while the risk of electronic cigarette use was similar across all racial and ethnic groups. This finding indicates that electronic cigarettes may be equally appealing or accessible across diverse racial and ethnic backgrounds. The higher risk among White adolescents for conventional cigarette use may be attributed to cultural norms, peer influences, or targeted marketing practices that differ from those affecting electronic cigarette use.

With a narrow age range (one year) of the cohort, age did not significantly impact the initiation of either electronic or conventional cigarette use. This suggests that factors other than age are more critical in influencing tobacco use initiation in early adolescence. The uniformity of risk across the age range studied highlights the importance of early prevention efforts, as susceptibility to tobacco use appears consistent from a young age.

Gender differences were also notable; male gender was a risk factor for conventional cigarette use, but both genders were equally likely to initiate electronic cigarette use. This indicates that electronic cigarettes may have a broader appeal across genders compared to conventional cigarettes. The traditional association of smoking with masculinity may explain the higher risk among males for conventional cigarette use, while the more modern and gender-neutral image of electronic cigarettes makes them attractive to both male and female adolescents.

### 5.1. Implications

Our findings underscore the importance of incorporating comprehensive screening for tobacco use in adolescents, considering both conventional and electronic cigarettes. Healthcare providers should routinely screen for tobacco use and assess the associated social determinants identified in this study. Early identification of at-risk adolescents can facilitate timely interventions to prevent tobacco use initiation.

Prevention programs should be tailored to address the specific risk factors identified, such as financial difficulties and family structure, while also recognizing the universal appeal of electronic cigarettes. Interventions targeting economic stressors and promoting family stability can help reduce the initiation of conventional cigarette use. Simultaneously, broad-based educational campaigns and policies that restrict access to electronic cigarettes are necessary to mitigate their impact.

The differential risk factors for conventional and electronic cigarette use emphasize the need for targeted prevention efforts. Conventional cigarette use remains influenced by financial difficulties, household income, and gender, suggesting that interventions should focus on addressing economic stressors and providing gender-specific support. Programs designed to alleviate financial hardship, such as economic assistance and stress management resources, could be effective in reducing conventional cigarette use among financially distressed adolescents. Additionally, gender-specific approaches that challenge the association of smoking with masculinity could help lower the risk for male adolescents.

In contrast, the uniform risk across various demographics for electronic cigarette use necessitates broad-based prevention strategies that encompass all adolescents, regardless of their background. Public health campaigns should aim to raise awareness about the risks associated with electronic cigarettes across all racial, ethnic, and socioeconomic groups. School-based programs that educate students about the dangers of electronic cigarettes and promote healthy alternatives to tobacco use are essential. Furthermore, policies that restrict the marketing and accessibility of electronic cigarettes to minors could help curb their widespread use.

Community-Based Participatory Research (CBPR) interventions have been applied for prevention of youth tobacco use, including both combustible products and electronic cigarettes, by fostering high levels of trust and efficiency in addressing community-specific needs. Such CBPR strategies involve a collaborative approach where researchers work closely with community members, stakeholders, and organizations throughout the research process. This partnership ensures that the interventions are culturally relevant, contextually appropriate, and tailored to the unique challenges and strengths of the community. By actively engaging the community in the design, implementation, and evaluation of the interventions, CBPR enhances the credibility and acceptability of the prevention strategies. Successful CBPR interventions for tobacco prevention often include education campaigns, peer-led programs, and community-driven policy advocacy. These strategies leverage the trust and insights of community members to effectively address the social determinants of tobacco use, such as stress, social norms, and targeted marketing by the tobacco industry. Moreover, CBPR approaches can better address the nuances of electronic cigarette use, which has distinct patterns and health implications compared to traditional tobacco products. By prioritizing the voices and experiences of the community, these interventions are more likely to be sustainable, equitable, and effective in reducing tobacco use among diverse populations, particularly in marginalized groups who may face additional barriers due to systemic inequities.

### 5.2. Limitations

Our study has several limitations. The data is observational, with omitted variables such as wealth. Longer term longitudinal studies with more comprehensive data collection are needed to confirm these findings and explore potential causal pathways. The narrow age range of the participants at baseline may restrict the generalizability of the findings to older adolescents. Our results are specific to a cohort of children followed from age nine, and the social determinants of tobacco use initiation may differ in older age groups. The short follow-up of the study also may have resulted in low incidence and low variation of the outcomes. Future research should include a broader age range as well as a longer follow-up to capture the developmental variations in tobacco use behaviors. Additionally, the reliance on self-reported data for tobacco use may have introduced reporting biases. Adolescents may underreport or misreport their tobacco use. Reports of social circumstances may also have some measurement bias. All of these may have led to potential inaccuracies in the results. Employing multiple data sources and verification methods could enhance the reliability of future studies. While we identified associations between social determinants and tobacco use initiation, we cannot establish causality.

## 6. Conclusion

In conclusion, our study highlights distinct social determinants for the early initiation of electronic and conventional cigarette use among U.S. adolescents. While financial difficulties, household income, family structure, and gender play significant roles in conventional cigarette use, electronic cigarette use appears to transcend these traditional social patterns. These findings call for a nuanced approach to tobacco prevention, addressing the unique challenges posed by electronic cigarettes and targeting at-risk groups effectively. Further research is needed to explore the long-term implications of these trends and to develop robust interventions to protect adolescent health.

## Figures and Tables

**Figure 1.**
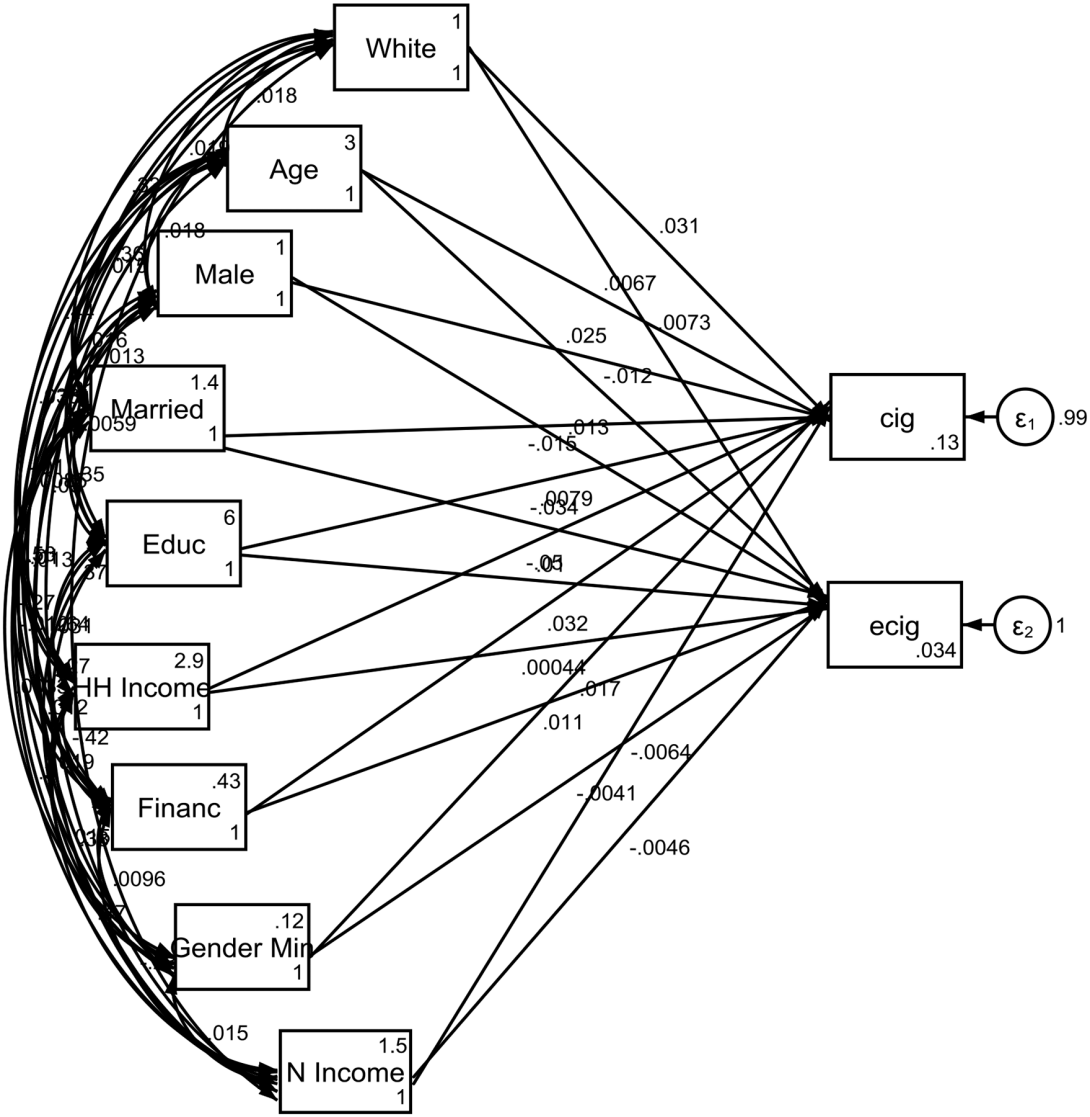
Summary of structural equation model (SEM)

**Table 1.** Summary of structural equation model (SEM)

Outcome	Predictor	B	SE	95% CI		p
Conventional Cigarette						
	Neighborhood income / 50000	−0.006	0.011	−0.027	0.015	0.548
	HH Income	−0.050	0.015	−0.080	−0.020	0.001
	Non-Latino White	0.031	0.011	0.010	0.051	0.004
	Financial difficulty (n)	0.032	0.010	0.012	0.052	0.002
	Age (10)	0.007	0.009	−0.011	0.025	0.466
	Male	0.025	0.009	0.007	0.043	0.006
	Married household	−0.015	0.011	−0.037	0.007	0.170
	Gender minority	0.017	0.009	−0.001	0.035	0.062
	Parental education	0.008	0.012	−0.016	0.032	0.517
	Intercept	0.131	0.066	0.001	0.261	0.048
						
Electronic Cigarette						
	Neighborhood income / 50000	−0.005	0.011	−0.026	0.016	0.669
	HH Income	0.000	0.016	−0.031	0.032	0.978
	Non-Latino White	0.007	0.011	−0.013	0.028	0.488
	Financial difficulty (n)	0.011	0.010	−0.009	0.031	0.268
	Age (10)	−0.012	0.009	−0.030	0.006	0.176
	Male	0.013	0.009	−0.005	0.031	0.159
	Married household	−0.034	0.011	−0.056	−0.012	0.003
	Gender minority	−0.004	0.009	−0.022	0.014	0.654
	Parental education	0.010	0.012	−0.014	0.035	0.399
	Intercept	0.034	0.066	−0.096	0.163	0.609
